# Long noncoding RNAs and their link to cancer

**DOI:** 10.1016/j.ncrna.2020.04.003

**Published:** 2020-05-13

**Authors:** Justine M. Grixti, Duncan Ayers

**Affiliations:** a Institute of Integrative Biology, Faculty of Health & Life Sciences, University of Liverpool, Liverpool, L69 6ZB, UK, United Kingdom; b Centre for Molecular Medicine and Biobanking, University of Malta, Msida, MSD2080, Malta; c Faculty of Biology, Medicine and Health Sciences, The University of Manchester, Manchester, M13 9PL, UK, United Kingdom

**Keywords:** lncRNA, Cancer, Tumour, Long noncoding RNA, Biomarkers

## Abstract

The central dogma of molecular biology, developed from the study of simple organisms such as *Escherichia coli*, has up until recently been that RNA functions mainly as an information intermediate between a DNA sequence (gene), localized in the cell nucleus, serving as a template for the transcription of messenger RNAs, which in turn translocate into the cytoplasm and act as blueprints for the translation of their encoded proteins. There are a number of classes of non-protein coding RNAs (ncRNAs) which are essential for gene expression to function. The specific number of ncRNAs within the human genome is unknown. ncRNAs are classified on the basis of their size. Transcripts shorter than 200 nucleotides, referred to as ncRNAs, which group includes miRNAs, siRNAs, piRNAs, etc, have been extensively studied. Whilst transcripts with a length ranging between 200 nt up to 100 kilobases, referred to as lncRNAs, make up the second group, and are recently receiving growing concerns. LncRNAs play important roles in a variety of biological processes, regulating physiological functions of organisms, including epigenetic control of gene regulation, transcription and post-transcription, affecting various aspects of cellular homeostasis, including proliferation, survival, migration and genomic stability. LncRNAs are also capable of tuning gene expression and impact cellular signalling cascades, play crucial roles in promoter-specific gene regulation, and X-chromosome inactivation. Furthermore, it has been reported that lncRNAs interact with DNA, RNA, and/or protein molecules, and regulate chromatin organisation, transcriptional and post-transcriptional regulation. Consequently, they are differentially expressed in tumours, and they are directly linked to the transformation of healthy cells into tumour cells. As a result of their key functions in a wide range of biological processes, lncRNAs are becoming rising stars in biology and medicine, possessing potential active roles in various oncologic diseases, representing a gold mine of potential new biomarkers and drug targets.

## 1 Introduction to lncRNAs

**Fig. 1:**
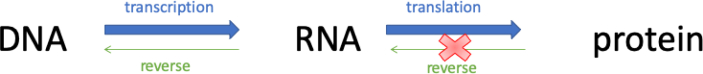
The central dogma of molecular biology.

The central dogma of molecular biology, developed from the study of simple organisms such as *Escherichia coli*, has up until recently been that RNA functions mainly as an information intermediate between a DNA sequence (gene), localized in the cell nucleus, serving as a template for the transcription of messenger RNAs, which in turn translocate into the cytoplasm and act as blueprints for the translation of their encoded proteins [1] (Fig. 1).

Gene expression is required for all aspects of life, and its regulation defines development and homeostasis of all cells, tissues, and organisms. There are a number of classes of non-protein coding RNAs (ncRNAs) which are essential for gene expression to function. These include small nuclear RNAs (snRNAs), mainly involved in mRNAs splicing events; transfer RNAs (tRNAs) which are responsible for specifically recognising three-nucleotide sequences of mRNAs, decoding the mRNA sequence into peptide or protein, and recruiting amino acids into ribosomes in the correct order. The most abundant cellular RNA molecules are represented by ribosomal RNAs (rRNAs), forming the framework of ribosomes. snRNAs, tRNAs, rRNAs are referred to as housekeeping RNAs and are constitutively expressed and are essential for normal cellular function [1].

Next generation sequencing methods and progress in transcriptome analysis have led to the discovery that up to 70% of the human genome is transcribed into RNA, however, only up to 2% of this serves as blueprints for proteins [[2], [3], [4], [5], [6]]. Moreover, the number of protein coding genes has remained quite steady during evolution in metazoan (G value paradox), whereas the size of genomes tends to increase. The advent of tiling resolution genomic microarrays and whole genome and transcriptome sequencing technologies showed that the human transcriptome is more complex than a collection of protein-coding genes and their splice variants; showing extensive antisense, overlapping and non-coding (ncRNA) expression [3,7]. In fact, 46% of the human genome, making up the largest part, consists of repetitive elements (such as transposons), and these have probably been the driving forces of evolution [7,8]. Moreover, it is also worth mentioning that in most cases transposons do not code for proteins and have been recently discovered to be related to cancer processes [9,10].

The term ncRNA is commonly used to refer to RNA which does not encode a protein. However, this does not imply that such RNA molecules do not contain any information and serve no function. Traditionally, it has been assumed that most genetic information is transacted by proteins. Recent evidence through the development of new techniques have revolutionised the molecular world and have shown that the majority of the mammalian and other complex organisms’ genomes is in fact transcribed into ncRNAs, which appear to comprise a hidden layer of internal signals controlling various levels of gene expression in physiology and development, including chromatin structure, epigenetic memory, transcription, RNA splicing, editing, translation and turnover [11].

## 2 lncRNAs

The specific number of ncRNAs within the human genome is unknown. ncRNAs are classified on the basis of their size. Transcripts shorter than 200 nucleotides, referred to as ncRNAs, which group includes miRNAs, siRNAs, piRNAs, etc, have been extensively studied. Whilst transcripts with a length ranging between 200 nt up to 100 kilobases, referred to as lncRNAs, make up the second group, and are recently receiving growing concerns. The latter transcripts lack a significant open reading frame [6,7,12].

Long non-coding RNAs (lncRNAs), encompassing nearly 30,000 different transcripts in humans, represent the most prevalent and functionally diverse class of ncRNAs [11]. There is no universal definition based on biological argumentation. Certain groups argue that lncRNAs may be classified into antisense, intergenic, overlapping, intronic, bidirectional, and processed subtypes, depending on the transcription position and direction in relation to other genes [13,14].. However, the most commonly used definition, an arbitrary one indeed, is based on the threshold of 200 nucleotides (nt) of RNA length [11] a lack of protein-coding potential and often harbour a poly-A tail and can be spliced, similar to mRNAs [1]. Conventionally, this divides RNAs into two groups; the lncRNAs which are >200 nt in length, and the remaining ones, referred to as “small” RNAs which are therefore <200 nt in length. The latter group includes many different RNAs, such as microRNAs (miRNAs), small nucleolar RNAs (snoRNAs), piwiRNAs (piRNAs) [11]. [15] attempted to distinguish between lncRNAs and small ncRNAs, by defining the former group as those ncRNAs which function either as primary or spliced transcripts, independent of extant known classes of small ncRNAs [3,15]. Such a definition places ncRNAs such as *BC1* and *snaR* in the lncRNA database, even though these are less than or close to 200 nt in length [15].

LncRNas are observed in a large diversity of species, including animals [16], plants [17], yeast [18], prokaryotes [19], and even viruses [3,20]. However, lncRNAs have been poorly conserved among different species when compared with the well-studied RNAs (such as mRNAs, miRNAs, snoRNAs). This in turn has invoked uncertainty as to whether a given lncRNA is function at all. Otherwise, the fact that there is poor interspecies conservation may convey functional species-specific characteristics. In addition, lncRNAs are usually low expressed [21,22], making them look more as transcriptional noise [11].

LncRNAs play important roles in a variety of biological processes, regulating physiological functions of organisms, including epigenetic control of gene regulation, transcription and post-transcription [5,6], affecting various aspects of cellular homeostasis, including proliferation, survival, migration and genomic stability. LncRNAs are also capable of tuning gene expression and impact cellular signalling cascades [23], play crucial roles in promoter-specific gene regulation, and X-chromosome inactivation [1]. Furthermore, it has been reported that lncRNAs interact with DNA, RNA, and/or protein molecules, and regulate chromatin organisation, transcriptional and post-transcriptional regulation [23]. Consequently, they are found to be differentially expressed in tumours, and they are directly linked to the transformation of healthy cells into tumour cells. As a result of their key functions in a wide range of biological processes, lncRNAs are becoming rising stars in biology and medicine, possessing potential active roles in various oncologic diseases, representing a gold mine of potential new biomarkers and drug targets [3,23,24].

Research is also showing that lncRNAs are deregulated in a number of human cancers, and their aberrant expression leads to cell proliferation, tumour initiation, growth and metastasis of cancer cells [[25], [26], [27], [28]]. More specifically [24], reported that they have identified 707 potential cancer-related lncRNAs, which act as scaffolds, interacting physically with other RNA species, resulting in a direct impact on cell signalling cascades. In this chapter, we seek to understand the link between cellular processes influenced by lncRNAs to the hallmarks of cancers [[3], [4], [5]]. This should serve to stimulate new research directions and therapeutic options, where lncRNAs can serve the purpose of novel prognostic markers, and therapeutic agents. However, even though the functional classification and link of lncRNAs to cancer is well-established, further studies are required so as to obtain a clearer characterisation with respect to phenotypic outputs, to suitably identify candidates which enable the development of new therapeutic strategies, together with the design of novel diagnostic approaches [13].

### 2.1 lncRNA and their link to cancer

A broad definition of cancer, also referred to as malignancy, is an abnormal and uncontrolled growth of cells, with the potential of invading or spreading of the affected cells to other parts of the body [29]. Cancer is primarily caused by genetic alteration which result in the deregulation of the gene networks that are responsible for the maintenance of cellular homeostasis, resulting due to interactions of somatic and germline mutations with various environmental factors [5]. There are more than 100 types of cancer, categorised according to the tissue of origin [30]. As a result, symptoms of cancer vary considerably with the type of tissue involved, location of origin, and type of genetic alteration causing the disease [29]. Research has pinpointed genetic alterations as being the main culprit behind this deadly disease. Several lifestyle and environmental related factors, including smoking, physical inactivity, high body fat, alcohol and caffeine intake, exposure to ultraviolet radiation, poor nutrition and high cholesterol intake diet, and use of aspirin [29,31], may also increase the risk of transforming normal cells to cancerous cells, altering the expression, at least in part, of various genes related to cellular proliferation and differentiation.

**Fig. 2:**
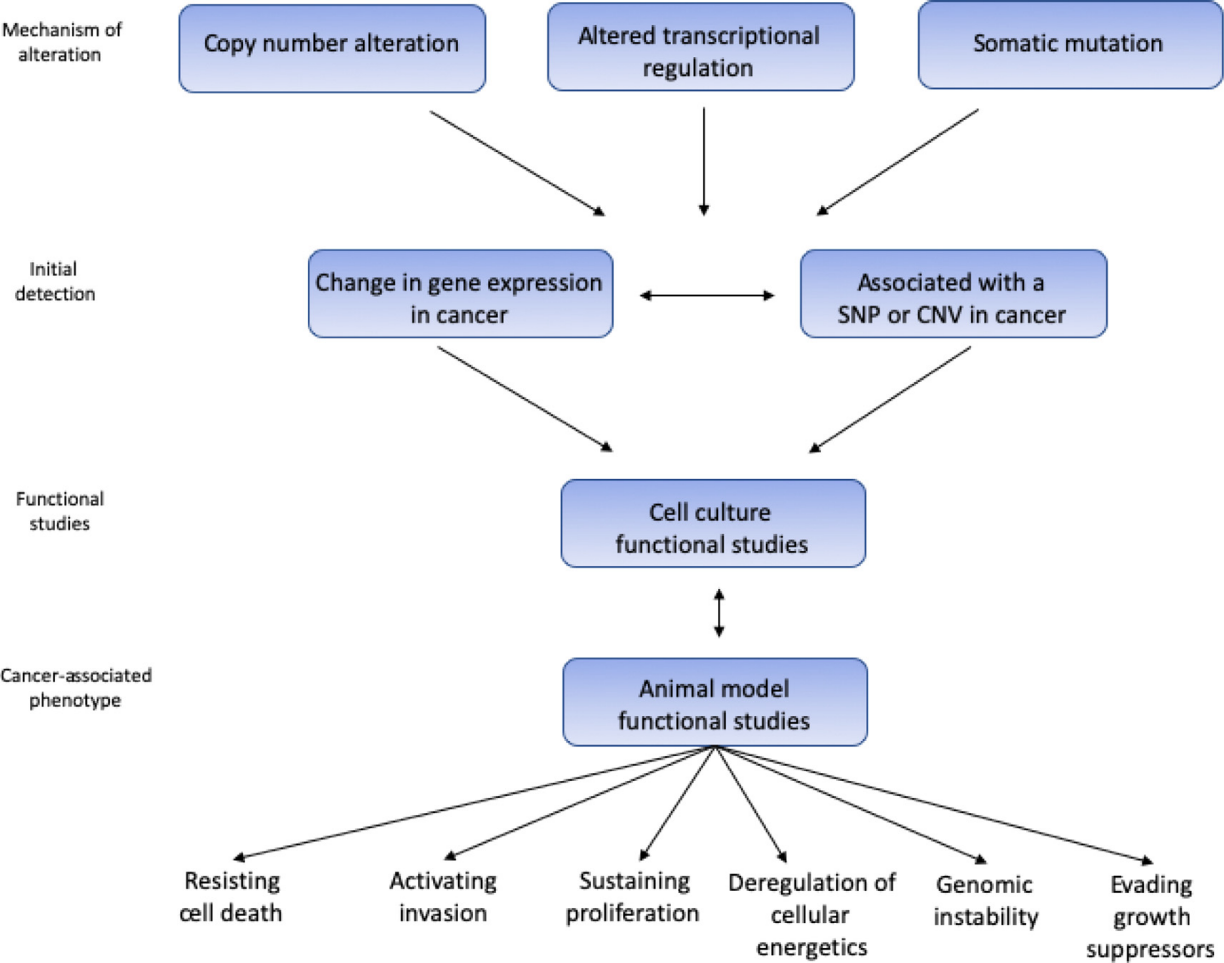
The various ways lncRNAs contribute to the hallmarks of cancer. Adopted from Ref. [5].

Several studies, particularly with the recent application of next-generation sequencing to a growing number of cancer transcriptomes, comparing malignant cells with their corresponding normal cells have revealed that many transcription factors, post-transcriptional regulators such as RNA binding proteins, microRNAs, and lncRNAs are crucial regulators for promoting or inhibiting tumour development [6,12,29]. LncRNAs have been gaining significant attention in terms of regulating the neoplastic transformation and progression [29], as well as being involved in the regulation of various cellular functions, including proliferation, migration, and DNA stability [29,32] even though only a few of these have been functionally characterised [12]. Fig. 2 below depicts the various ways lncRNAs are linked to the hallmarks of cancer.

[5] reported that prostate cancer associated 3 (PCA3, also referred to as DD3) and prostate-specific transcript 1 (PCGEM1) were the first lncRNAs that were associated with cancer because of their aberrant expression, found to have differential display analysis of prostate tumours and normal tissue [5]. PCA3 is currently used as a prostate cancer biomarker [33].

[6] have reported that a number of lncRNAs are involved in important processes of breast cancer (BC), including 1. Promotion and proliferation of BC or apoptosis inhibition of BC (lncRNAs include: H19, SRA, LINC01296); 2. Promotion of drug resistance in BD cells (lncRNAs include: UCA1, CRALA, lnc-ATB); 3. Promotion of invasion and metastasis of BC cells (lncRNAs include: HOTAIR, MALAT1, CCAT2) [5,6]. Some other lncRNAs have been shown to inhibit these processes [6]. So much so that lncRNAs have the potential to serve as biomarkers in certain cancer types [29], more specifically in those malignancies where the alternation of these ncRNAs are associated with cancer development, progression, and metastasis [34]. Moreover, a unique pattern of expression of some lncRNAs in specific types of cancer has made them attractive targets for drug development [35]. The following table (Table 1) highlights the expression of lncRNAs in different types of cancer.

**Table 1:** lncRNAs in different types of cancers.

Cancer types	lncRNAs	Description	References
Colorectal and prostate cancers	CCAT2, PCAT-1	Over-expressed	[[36], [37], [38], [39]]
Non small cell lung cancer	MALATI, HOTAIR, CCAT2, AK126698	Cancer progression, metastasis and invasion	[[40], [41], [42], [43]]
Liver cancer	HULC	Promotes tumor angiogenesis	[44]
Breast cancer	ATB	Over-expression is associated with the highly metastatic phenotype	[6,45]
Gastric cancer	AP001631.9	Promotes cell migration	[46]
Prostate cancer	DRAIC and PCAT29	Over-expression inhibit the migration and invasion of cancer cells	[[47], [48], [49]]
Hepatocellular carcinoma	HNF1A-AS1	Stimulates proliferation and suppressor of apoptosis	[50,51]
Pancreatic ductal adenocarcinoma	lncRNA-MIR31HG	Upregulated expression	[[52], [53], [54]]

Moreover [12,12], have reported that the upregulation of certain lncRNAs, including HOTAIR, MALAT1, CCAT2, and the downregulation of LOC285194, UC.388, and LET have been implicated in promoting the metastasis of colorectal cancer (CRC). However, the biological and pathological functions of their mechanism remains a field to be studied, since most lncRNAs which were expected to be prognostic and predictive in cancer patients have unfortunately failed to perform these functions when tested *in vivo* [12].

### 2.2 Mechanism(s) of lncRNA action

A number of studies using next generation sequencing have revealed that a significant portion of the mutation associated with cancer development lies within the non-coding region of the human genome, which mutation has a particular effect on the expression of lncRNAs, which in turn may regulate various cancer phenotypes by interacting with DNA, RNA, and proteins [35]. Research has demonstrated that a number of lncRNAs have been reported to be aberrantly expressed in tumours, showing crosstalk with key cancer-related signalling pathways [55], with the main mechanism(s) of action of lncRNA on cancer cells regulate the expression of target genes in the following ways [29]:

i. Facilitating combinatorial actions of different transcription factors
ii. Removing transcription factors and other regulatory protein from chromatin
iii. Recruiting chromatin modifiers in *cis* and *trans* genes
iv. acting as scaffolds bringing multiple proteins together, forming ribonucleoprotein complexes inducing histone modification
v. Interact with DNA methyltransferase enzymes through other protein mediators, regulating DA methylation in both *cis* and *trans* genes

**Fig. 3:**
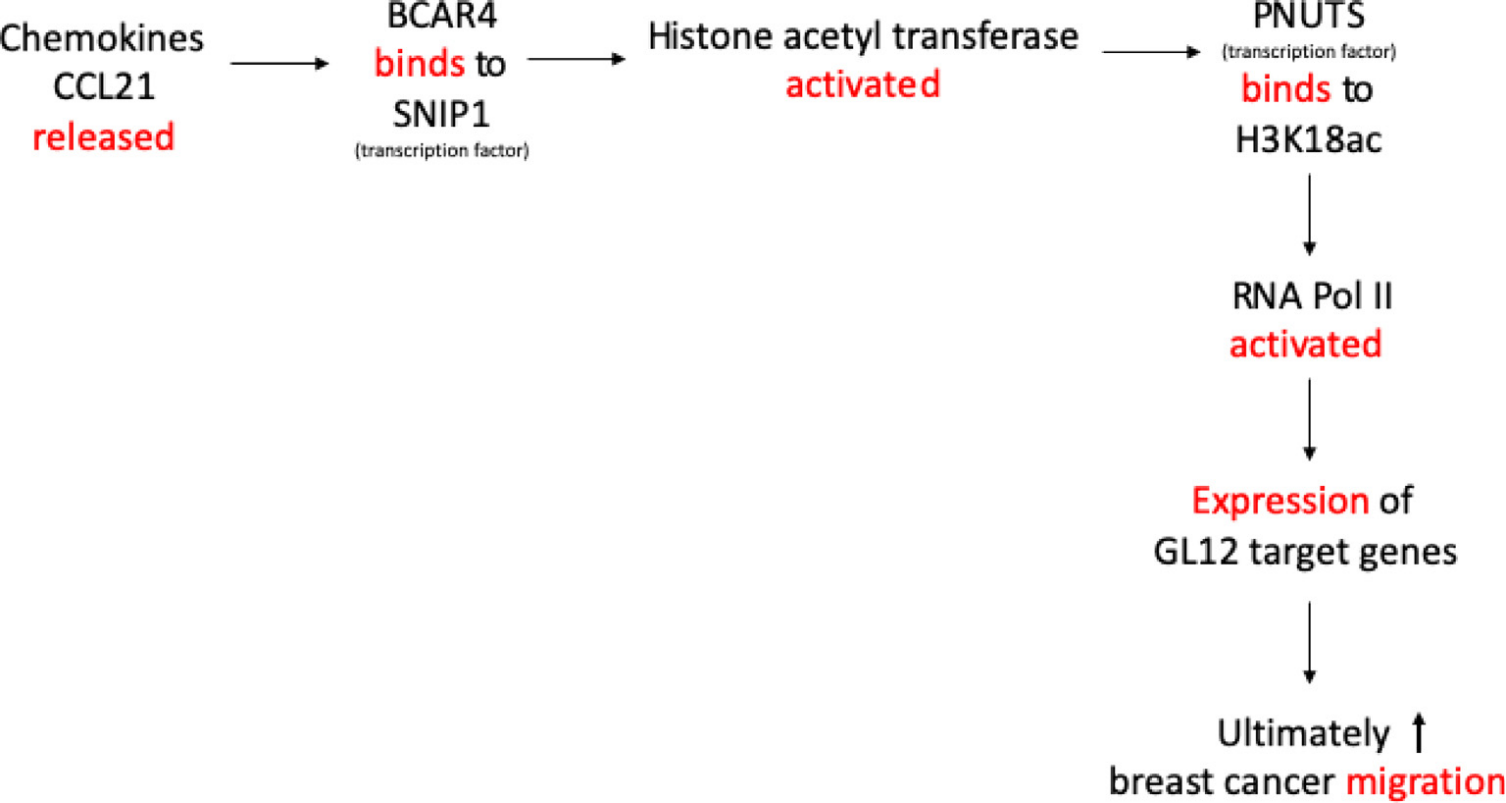
Mechanism depicting how the expression of lncRNA BCAR4 is associated *with advanced breast cancer.*

Moreover, lncRNAs are significantly associated with the growth, survival, migration, and angiogenesis of a number of cancer cell types by transcriptionally or posttranscriptionally regulating the epigenetic regulators/modifiers [29,55]. The following (Fig. 3) depicts the role of lncRNA, BCAR4, in the metastasis of breast cancer via chemokine-induced binding of BCAR4 to two transcription factors having extended regulatory consequences.

Furthermore, Pei-fen [55] have reported that BCAR4 wires up Hippo and Hedgehog signalling to reprogram glucose metabolism. Besides, plasma lipid-associated lncRNA has been shown to regulate normoxic hypoxia-inducible factor 1∝ (HIF-1∝) stabilisation, as depicted in Table 2 below.

**Table 2:** Cytoplasmic lncRNAs in cancer signalling pathways. Table adapted from Refs. [55]].

Gene	Main distribution	Related signalling pathway
LINK-A	Triple - negative breast tumour	HIF - 1 α; PI3 K/A KT
MAYA	Majority of human solid tumours	Hippo - YA P
Lnc-DC	Dendritic cells	STAT3
NKILA	Normal breast epitheliaor non -invasivebreast tumours	N F - KB
ACOD1	Most of the cells and organs	GOT 2 metabiolic pathw
Lnc- Lsm 3 b	Immune cells and organs	IFN1

### 2.3 Role of lncRNAs as tumour suppressors

**Fig. 4:**
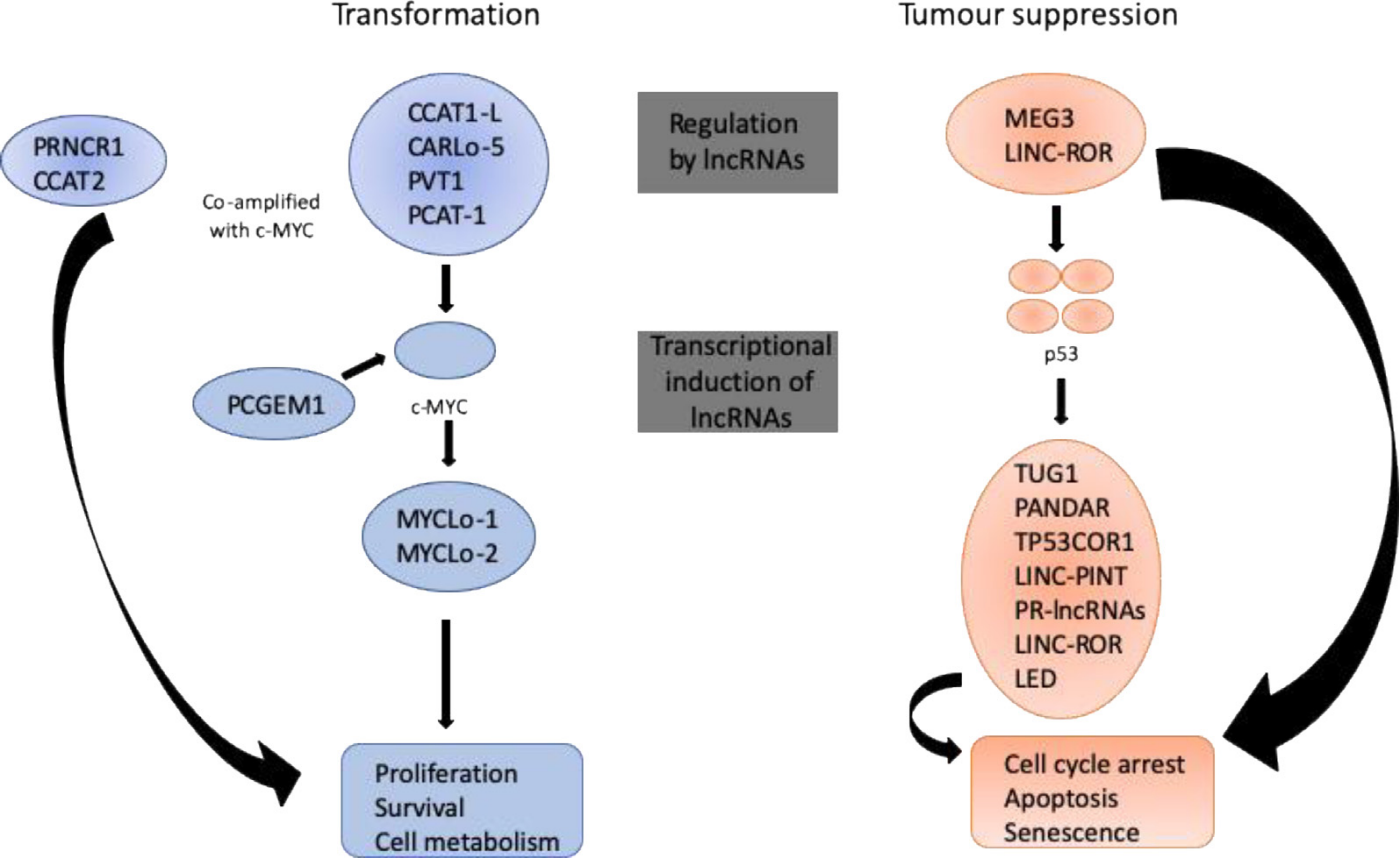
A number of lncRNAs regulate c-MYC or p53 9MEG3, LINK-ROR) by affecting their expression, protein levels or activity. Hence, lncRNAs form part of the c-MYC oncogenic and p53 tumour suppressor networks.

LncRNAs can also act as tumour suppressors. Genome-wide studies have revealed that transcription factors, such as p53 [32,56], MYC [57,58] or the oestrogen receptor [59] specifically regulate the expression of a number of lncRNAs. One of the major tumour suppressor proteins and preserver of cellular homeostasis, identified so far is p53, playing a vital role in genomic stability, regulating its downstream target genes by binding specifically to p53 response element (p53RE). Research has shown that p53RE lies on the genomic region that encodes lncRNAs, suggesting a possible role of lncRNAs as tumour suppressors. For example, following DNA damage or oncogenic stress, the transcription factor p53 initiates a tumour suppressor program which involves the induction of many genes, including lncRNAs, and as shown in Fig. 4, some of these lncRNAs are direct transcriptional targets of p53.

**Fig. 5:**
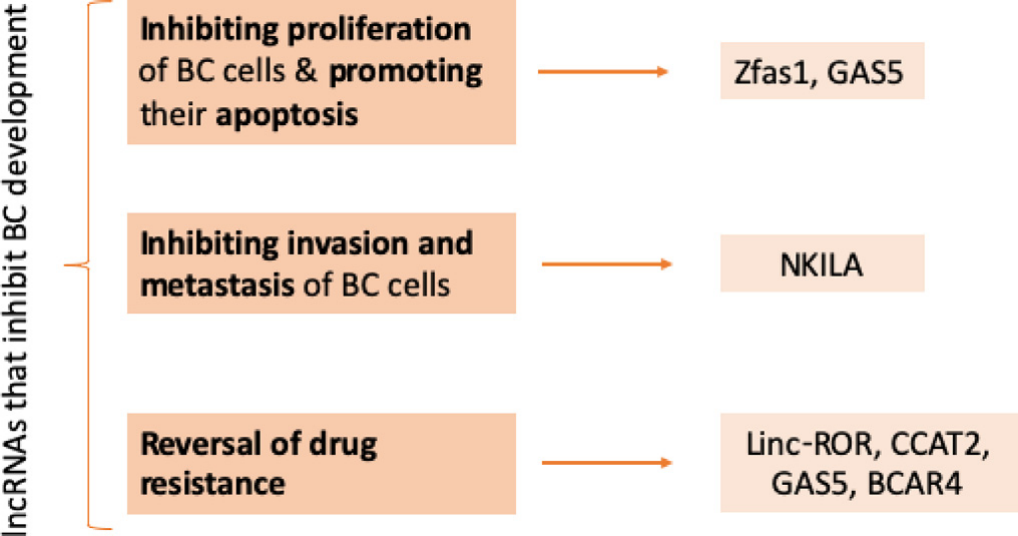
lncRNAs inhibiting BC development.

[6] have reported that there are a number of lncRNAs, as shown in Fig. 5 below, that are related with inhibiting the development of BC [6].

## 3 Conclusion

Given the important of lncRNAs in controlling important cellular processes, it is sound to say that similar to protein-coding regions of the human genome, genetic regions encoding lncRNAs play equally important roles in regulating the malignant transformation and progression. With the improvement of research methods such as the development of gene array technologies and high-throughput sequencing technologies, more categories of lncRNAs are expected to be discovered, which technologies will allow for the effective understanding of their complex mechanism/s of action, and eventually using recent CRISPR/CAS gene editing technologies to play certain roles in developing lncRNAs as tumour suppressor therapies, delivering novel and alternative treatment strategies for targeting cancer associated lncRNAs.

## References

[1] Kretz S.H.M. (2016). Non-Coding RNAs in Colorectal Cancer.

[2] Stein L.D. (2004). Human genome: end of the beginning. Nature.

[3] Gibb E.A., Brown C.J., Lam W.L. (2011). The functional role of long non-coding RNA in human carcinomas. Mol. Canc..

[4] Gutschner T., Diederichs S. (2012). The hallmarks of cancer: a long non-coding RNA point of view. RNA Biol..

[5] Huarte M. (2015). The emerging role of lncRNAs in cancer. Nat. Med..

[6] Bin X., Hongjian Y., Xiping Z., Bo C., Shifeng Y., Binbin T. (2018). Research progresses in roles of LncRNA and its relationships with breast cancer. Canc. Cell Int..

[7] Spizzo R., Almeida M.I., Colombatti A., Calin G.A. (2012). Long non-coding RNAs and cancer: a new frontier of translational research?. Oncogene.

[8] Cordaux R., Batzer M.A. (2009). The impact of retrotransposons on human genome evolution. Nat. Rev. Genet..

[9] Iskow R.C., McCabe M.T., Mills R.E., Torene S., Pittard W.S., Neuwald A.F., Van Meir E.G., Vertino P.M., Devine S.E. (2010). Natural mutagenesis of human genomes by endogenous retrotransposons. Cell.

[10] Sunami E., de Maat M., Vu A., Turner R.R., Hoon D.S. (2011). LINE-1 hypomethylation during primary colon cancer progression. PloS One.

[11] Ma L., Bajic V.B., Zhang Z. (2013). On the classification of long non-coding RNAs. RNA Biol..

[12] Chen D., Sun Q., Cheng X., Zhang L., Song W., Zhou D., Lin J., Wang W. (2016). Genome-wide analysis of long noncoding RNA (lncRNA) expression in colorectal cancer tissues from patients with liver metastasis. Cancer Med.

[13] Kung J.T., Colognori D., Lee J.T. (2013). Long noncoding RNAs: past, present, and future. Genetics.

[14] Peschansky V.J., Wahlestedt C. (2014). Non-coding RNAs as direct and indirect modulators of epigenetic regulation. Epigenetics.

[15] Amaral P.P., Clark M.B., Gascoigne D.K., Dinger M.E., Mattick J.S. (2011). lncRNAdb: a reference database for long noncoding RNAs. Nucleic Acids Res..

[16] Brown C.J., Hendrich B.D., Rupert J.L., Lafreniere R.G., Xing Y., Lawrence J., Willard H.F. (1992). The human XIST gene: analysis of a 17 kb inactive X-specific RNA that contains conserved repeats and is highly localized within the nucleus. Cell.

[17] Swiezewski S., Liu F., Magusin A., Dean C. (2009). Cold-induced silencing by long antisense transcripts of an Arabidopsis Polycomb target. Nature.

[18] Houseley J., Rubbi L., Grunstein M., Tollervey D., Vogelauer M. (2008). A ncRNA modulates histone modification and mRNA induction in the yeast GAL gene cluster. Mol. Cell..

[19] Bernstein H.D., Zopf D., Freymann D.M., Walter P. (1993). Functional substitution of the signal recognition particle 54-kDa subunit by its Escherichia coli homolog. Proc. Natl. Acad. Sci. U. S. A..

[20] Reeves M.B., Davies A.A., McSharry B.P., Wilkinson G.W., Sinclair J.H. (2007). Complex I binding by a virally encoded RNA regulates mitochondria-induced cell death. Science.

[21] van Bakel H., Nislow C., Blencowe B.J., Hughes T.R. (2010). Most "dark matter" transcripts are associated with known genes. PLoS Biol..

[22] Cabili M.N., Trapnell C., Goff L., Koziol M., Tazon-Vega B., Regev A., Rinn J.L. (2011). Integrative annotation of human large intergenic noncoding RNAs reveals global properties and specific subclasses. Genes Dev..

[23] Sanchez L.R., Borriello L., Entenberg D., Condeelis J.S., Oktay M.H., Karagiannis G.S. (2019). The emerging roles of macrophages in cancer metastasis and response to chemotherapy. J. Leukoc. Biol..

[24] Zhao T., Xu J., Liu L., Bai J., Xu C., Xiao Y., Li X., Zhang L. (2015). Identification of cancer-related lncRNAs through integrating genome, regulome and transcriptome features. Mol. Biosyst..

[25] Mercer T.R., Dinger M.E., Sunkin S.M., Mehler M.F., Mattick J.S. (2008). Specific expression of long noncoding RNAs in the mouse brain. Proc. Natl. Acad. Sci. U. S. A..

[26] Loewer S., Cabili M.N., Guttman M., Loh Y.H., Thomas K., Park I.H., Garber M., Curran M., Onder T., Agarwal S., Manos P.D., Datta S., Lander E.S., Schlaeger T.M., Daley G.Q., Rinn J.L. (2010). Large intergenic non-coding RNA-RoR modulates reprogramming of human induced pluripotent stem cells. Nat. Genet..

[27] Taft R.J., Pang K.C., Mercer T.R., Dinger M., Mattick J.S. (2010). Non-coding RNAs: regulators of disease. J. Pathol..

[28] Maruyama R., Shipitsin M., Choudhury S., Wu Z., Protopopov A., Yao J., Lo P.K., Bessarabova M., Ishkin A., Nikolsky Y., Liu X.S., Sukumar S., Polyak K. (2012). Altered antisense-to-sense transcript ratios in breast cancer. Proc. Natl. Acad. Sci. U. S. A..

[29] Rathinasamy B., Velmurugan B.K. (2018). Role of lncRNAs in the cancer development and progression and their regulation by various phytochemicals. Biomed. Pharmacother..

[30] Blackadar C.B. (2016). Historical review of the causes of cancer. World J. Clin. Oncol..

[31] Grixti J.M., O'Hagan S., Day P.J., Kell D.B. (2017). Enhancing drug efficacy and therapeutic index through cheminformatics-based selection of small molecule binary weapons that improve transporter-mediated targeting: a cytotoxicity system based on gemcitabine. Front. Pharmacol..

[32] Huarte M., Guttman M., Feldser D., Garber M., Koziol M.J., Kenzelmann-Broz D., Khalil A.M., Zuk O., Amit I., Rabani M., Attardi L.D., Regev A., Lander E.S., Jacks T., Rinn J.L. (2010). A large intergenic noncoding RNA induced by p53 mediates global gene repression in the p53 response. Cell.

[33] Hessels D., Klein Gunnewiek J.M., van Oort I., Karthaus H.F., van Leenders G.J., van Balken B., Kiemeney L.A., Witjes J.A., Schalken J.A. (2003). DD3(PCA3)-based molecular urine analysis for the diagnosis of prostate cancer. Eur. Urol..

[34] Martens-Uzunova E.S., Bottcher R., Croce C.M., Jenster G., Visakorpi T., Calin G.A. (2014). Long noncoding RNA in prostate, bladder, and kidney cancer. Eur. Urol..

[35] Schmitt A.M., Chang H.Y. (2016). Long noncoding RNAs in cancer pathways. Canc. Cell.

[36] Tomlinson I., Webb E., Carvajal-Carmona L., Broderick P., Kemp Z., Spain S., Penegar S., Chandler I., Gorman M., Wood W., Barclay E., Lubbe S., Martin L., Sellick G., Jaeger E., Hubner R., Wild R., Rowan A., Fielding S., Howarth K., Consortium C., Silver A., Atkin W., Muir K., Logan R., Kerr D., Johnstone E., Sieber O., Gray R., Thomas H., Peto J., Cazier J.B., Houlston R. (2007). A genome-wide association scan of tag SNPs identifies a susceptibility variant for colorectal cancer at 8q24.21. Nat. Genet..

[37] Eeles R.A., Kote-Jarai Z., Giles G.G., Olama A.A., Guy M., Jugurnauth S.K., Mulholland S., Leongamornlert D.A., Edwards S.M., Morrison J., Field H.I., Southey M.C., Severi G., Donovan J.L., Hamdy F.C., Dearnaley D.P., Muir K.R., Smith C., Bagnato M., Ardern-Jones A.T., Hall A.L., O'Brien L.T., Gehr-Swain B.N., Wilkinson R.A., Cox A., Lewis S., Brown P.M., Jhavar S.G., Tymrakiewicz M., Lophatananon A., Bryant S.L., Horwich A., Huddart R.A., Khoo V.S., Parker C.C., Woodhouse C.J., Thompson A., Christmas T., Ogden C., Fisher C., Jamieson C., Cooper C.S., English D.R., Hopper J.L., Neal D.E., Easton D.F., U. K. G. P. C. S. Collaborators, O. British Association of Urological Surgeons, Section of, U. K. P. S. Collaborators (2008). Multiple newly identified loci associated with prostate cancer susceptibility. Nat. Genet..

[38] Prensner J.R., Iyer M.K., Balbin O.A., Dhanasekaran S.M., Cao Q., Brenner J.C., Laxman B., Asangani I.A., Grasso C.S., Kominsky H.D., Cao X., Jing X., Wang X., Siddiqui J., Wei J.T., Robinson D., Iyer H.K., Palanisamy N., Maher C.A., Chinnaiyan A.M. (2011). Transcriptome sequencing across a prostate cancer cohort identifies PCAT-1, an unannotated lincRNA implicated in disease progression. Nat. Biotechnol..

[39] Ling H., Spizzo R., Atlasi Y., Nicoloso M., Shimizu M., Redis R.S., Nishida N., Gafa R., Song J., Guo Z., Ivan C., Barbarotto E., De Vries I., Zhang X., Ferracin M., Churchman M., van Galen J.F., Beverloo B.H., Shariati M., Haderk F., Estecio M.R., Garcia-Manero G., Patijn G.A., Gotley D.C., Bhardwaj V., Shureiqi I., Sen S., Multani A.S., Welsh J., Yamamoto K., Taniguchi I., Song M.A., Gallinger S., Casey G., Thibodeau S.N., Le Marchand L., Tiirikainen M., Mani S.A., Zhang W., Davuluri R.V., Mimori K., Mori M., Sieuwerts A.M., Martens J.W., Tomlinson I., Negrini M., Berindan-Neagoe I., Foekens J.A., Hamilton S.R., Lanza G., Kopetz S., Fodde R., Calin G.A. (2013). CCAT2, a novel noncoding RNA mapping to 8q24, underlies metastatic progression and chromosomal instability in colon cancer. Genome Res..

[40] Yang Y., Li H., Hou S., Hu B., Liu J., Wang J. (2013). The noncoding RNA expression profile and the effect of lncRNA AK126698 on cisplatin resistance in non-small-cell lung cancer cell. PloS One.

[41] Zhan Y., Zang H., Feng J., Lu J., Chen L., Fan S. (2017). Long non-coding RNAs associated with non-small cell lung cancer. Oncotarget.

[42] Zhao Z., Wang J., Wang S., Chang H., Zhang T., Qu J. (2017). LncRNA CCAT2 promotes tumorigenesis by over-expressed Pokemon in non-small cell lung cancer. Biomed. Pharmacother..

[43] Jiang C., Yang Y., Yang Y., Guo L., Huang J., Liu X., Wu C., Zou J. (2018). Long noncoding RNA (lncRNA) HOTAIR affects tumorigenesis and metastasis of non-small cell lung cancer by upregulating miR-613. Oncol. Res..

[44] Klec C., Gutschner T., Panzitt K., Pichler M. (2019). Involvement of long non-coding RNA HULC (highly up-regulated in liver cancer) in pathogenesis and implications for therapeutic intervention. Expert Opin. Ther. Targets.

[45] Shi S.J., Wang L.J., Yu B., Li Y.H., Jin Y., Bai X.Z. (2015). LncRNA-ATB promotes trastuzumab resistance and invasion-metastasis cascade in breast cancer. Oncotarget.

[46] Cai H., Ye X., He B., Li Q., Li Y., Gao Y. (2015). LncRNA-AP001631.9 promotes cell migration in gastric cancer. Int. J. Clin. Exp. Pathol..

[47] Malik R., Patel L., Prensner J.R., Shi Y., Iyer M.K., Subramaniyan S., Carley A., Niknafs Y.S., Sahu A., Han S., Ma T., Liu M., Asangani I.A., Jing X., Cao X., Dhanasekaran S.M., Robinson D.R., Feng F.Y., Chinnaiyan A.M. (2014). The lncRNA PCAT29 inhibits oncogenic phenotypes in prostate cancer. Mol. Canc. Res..

[48] Al Aameri R.F.H., Sheth S., Alanisi E.M.A., Borse V., Mukherjea D., Rybak L.P., Ramkumar V. (2017). Tonic suppression of PCAT29 by the IL-6 signaling pathway in prostate cancer: reversal by resveratrol. PloS One.

[49] Zhao D., Dong J.T. (2018). Upregulation of long non-coding RNA DRAIC correlates with adverse features of breast cancer. Noncoding RNA.

[50] Wang C., Mou L., Chai H.X., Wang F., Yin Y.Z., Zhang X.Y. (2017). Long non-coding RNA HNF1A-AS1 promotes hepatocellular carcinoma cell proliferation by repressing NKD1 and P21 expression. Biomed. Pharmacother..

[51] Ding C.H., Yin C., Chen S.J., Wen L.Z., Ding K., Lei S.J., Liu J.P., Wang J., Chen K.X., Jiang H.L., Zhang X., Luo C., Xie W.F. (2018). The HNF1alpha-regulated lncRNA HNF1A-AS1 reverses the malignancy of hepatocellular carcinoma by enhancing the phosphatase activity of SHP-1. Mol. Canc..

[52] Yang H., Liu P., Zhang J., Peng X., Lu Z., Yu S., Meng Y., Tong W.M., Chen J. (2016). Long noncoding RNA MIR31HG exhibits oncogenic property in pancreatic ductal adenocarcinoma and is negatively regulated by miR-193b. Oncogene.

[53] Duguang L., Jin H., Xiaowei Q., Peng X., Xiaodong W., Zhennan L., Jianjun Q., Jie Y. (2017). The involvement of lncRNAs in the development and progression of pancreatic cancer. Canc. Biol. Ther..

[54] Lv Y., Huang S. (2019). Role of non-coding RNA in pancreatic cancer. Oncol Lett.

[55] Fu P.F., Zheng X., Fan X., Lin A.F. (2019). Role of cytoplasmic lncRNAs in regulating cancer signaling pathways. J. Zhejiang Univ. - Sci. B.

[56] Sanchez Y., Segura V., Marin-Bejar O., Athie A., Marchese F.P., Gonzalez J., Bujanda L., Guo S., Matheu A., Huarte M. (2014). Genome-wide analysis of the human p53 transcriptional network unveils a lncRNA tumour suppressor signature. Nat. Commun..

[57] Hart J.R., Roberts T.C., Weinberg M.S., Morris K.V., Vogt P.K. (2014). MYC regulates the non-coding transcriptome. Oncotarget.

[58] Kim T., Jeon Y.J., Cui R., Lee J.H., Peng Y., Kim S.H., Tili E., Alder H., Croce C.M. (2015). Role of MYC-regulated long noncoding RNAs in cell cycle regulation and tumorigenesis. J. Natl. Cancer Inst..

[59] Chakravarty D., Sboner A., Nair S.S., Giannopoulou E., Li R., Hennig S., Mosquera J.M., Pauwels J., Park K., Kossai M., MacDonald T.Y., Fontugne J., Erho N., Vergara I.A., Ghadessi M., Davicioni E., Jenkins R.B., Palanisamy N., Chen Z., Nakagawa S., Hirose T., Bander N.H., Beltran H., Fox A.H., Elemento O., Rubin M.A. (2014). The oestrogen receptor alpha-regulated lncRNA NEAT1 is a critical modulator of prostate cancer. Nat. Commun..

